# Supplementary material for: A reference genome for the long-term kleptoplast-retaining sea slug Elysia crispata morphotype clarki
Source: G3 (Bethesda). 2023 Oct 10;13(12):jkad234. doi: 10.1093/g3journal/jkad234 (PMC10700116; doi:10.1093/g3journal/jkad234)
Supplement: jkad234_Supplementary_Data [file jkad234_supplementary_data.pdf]

Supporting Information for

A reference genome for the long-term kleptoplast-retaining sea slug *Elysia crispata* morphotype clarki

Katharine E. Eastman, Amanda L. Pendleton, Mearaj A. Shaikh, Thiti Suttiyut, Raeya Ogas, Paxton Tomko, Gregory Gavelis, Joshua R. Widhalm, and Jennifer H. Wisecaver

Correspondence: jwisecav@purdue.edu and jwidhalm@purdue.edu

This PDF file includes:
Figures S1 to S9

Other supporting materials for this manuscript include the following:
Tables S1 to S20 available through FigShare (https://doi.org/10.6084/m9.figshare.23635812.v1)

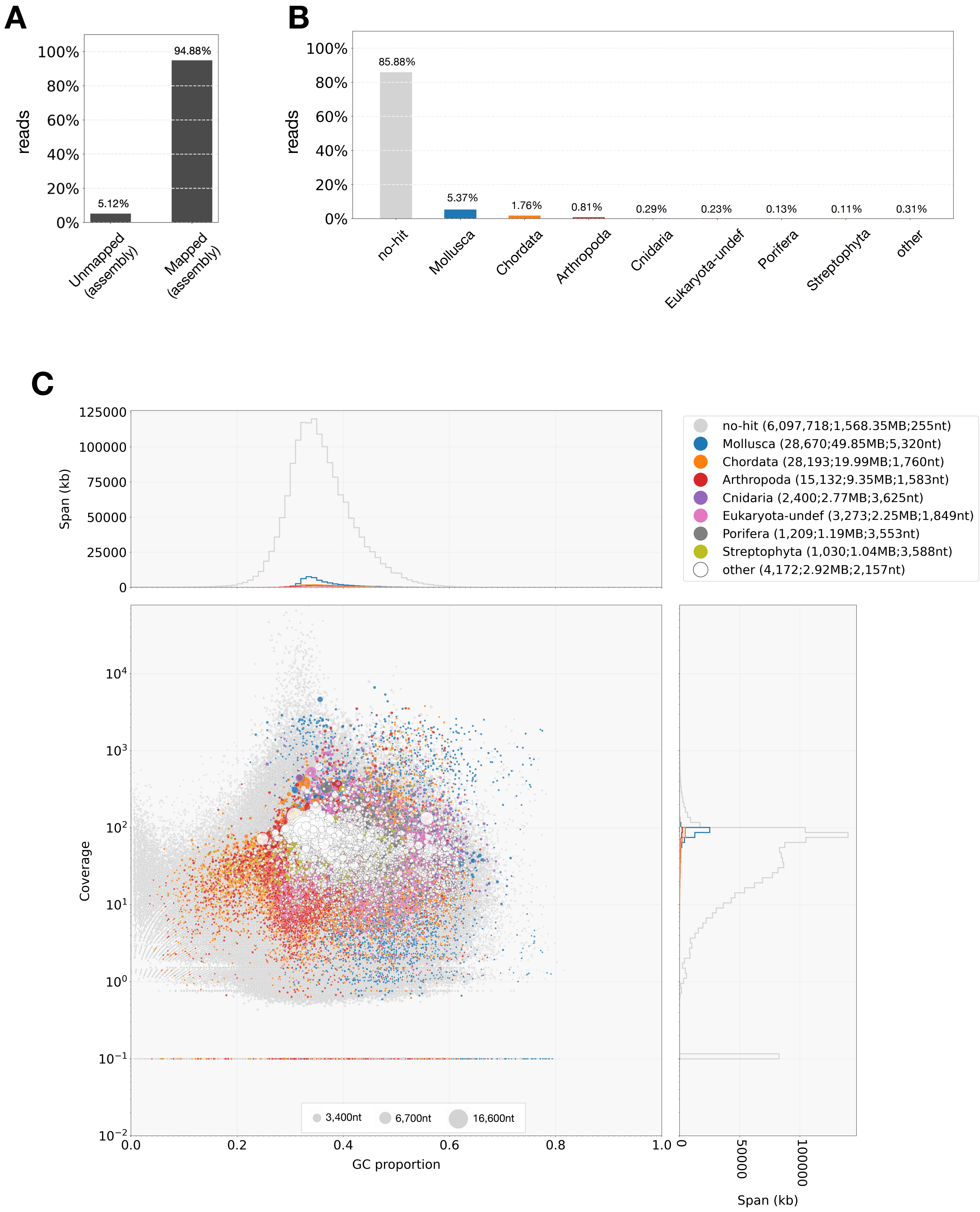

Figure S1. BlotTools summary output. A) Read coverage plots visualizing the proportion of Illumina reads that unmapped/mapped to the preliminary Illumina-only assembly. B) Read coverage plots showing the percentage of mapped reads by taxonomic group, as barcharts. C) Blobplots showing taxonomic breakdown of Illumina short-read assemblies. Colored circles indicate contigs with majority hits to protein and/or nucleotide sequences from Mollusca (blue), Chordata (orange), Arthropoda (red), Cnidaria (purple) Eukaryota undefined (pink), Porifera (dark green), Streptophyta (light green), and other (white). Contigs that could not be assigned to a taxonomic lineage are indicated by the grey circles. Contigs with majority hits to any non-eukaryotic lineage were excluded from further analysis, and reads that mapped to excluded contigs were also removed.

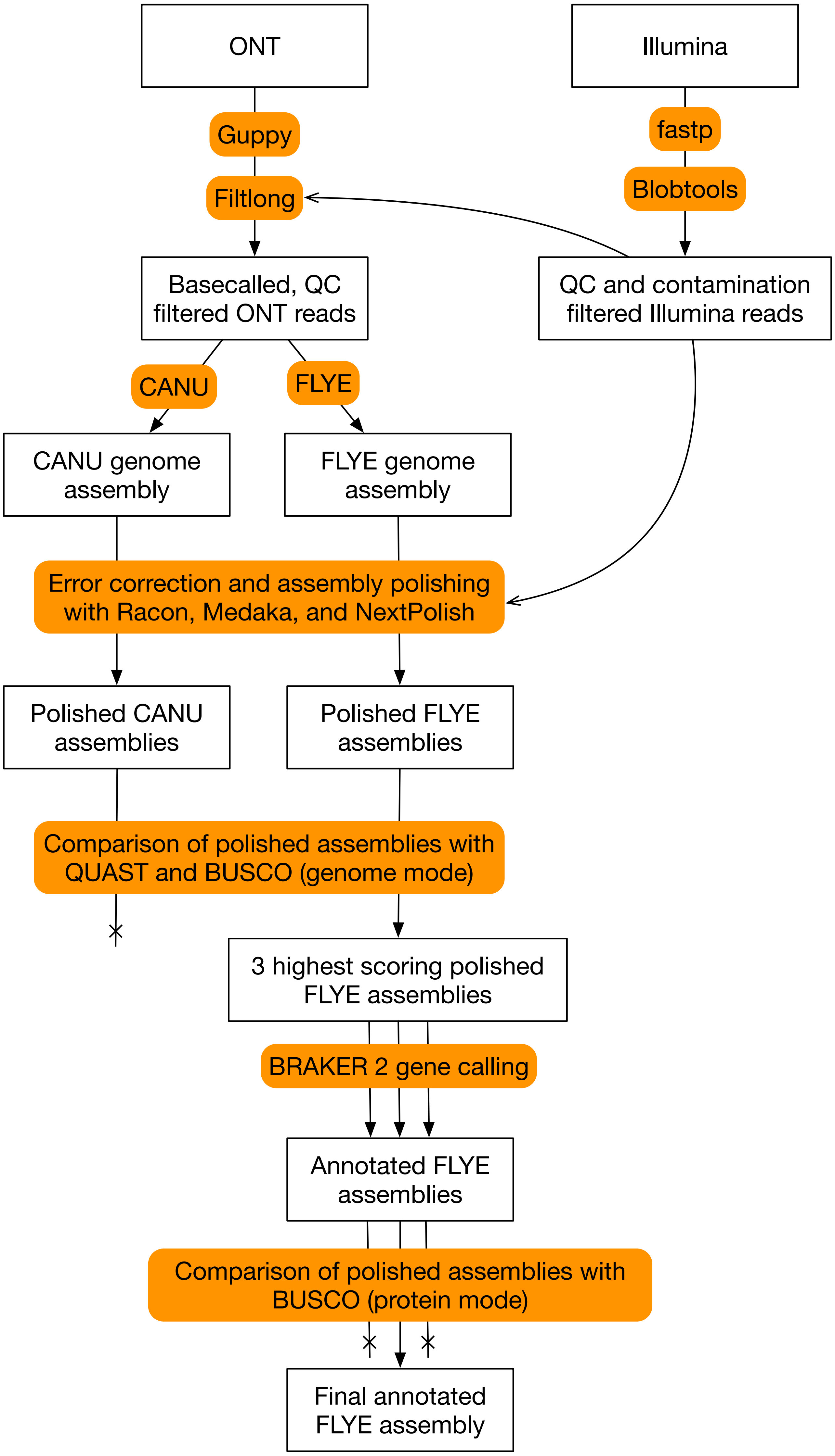

Figure S2. Genome assembly workflow. See methods for software versions and references.

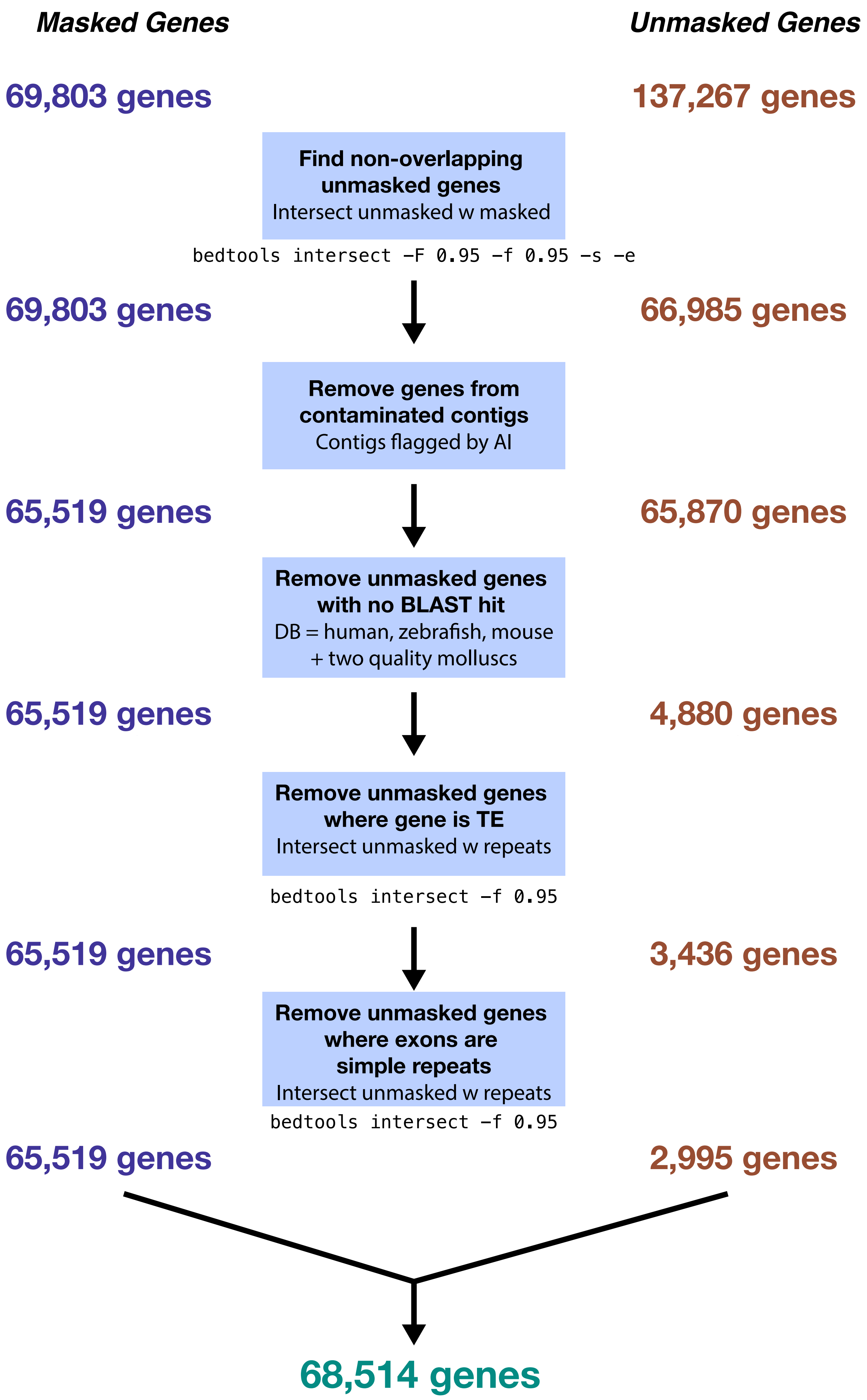

Figure S3. Curation of final gene model set. Gene models resulting from Braker annotation with and without the use of the masked reference assembly were independently filtered to retain the highest quality gene models. Unique gene models resulting from an unmasked Braker annotation were identified through coordinate intersection with the masked gene set, and were then required to have a BLAST hit to a gene model (database included human, zebrafish, mouse, and two mollusks), not intersect with a classified repeat element (LINE, SINE, LTR, etc.), and not have axons that were >50% simple sequence repeats. Gene models from both annotation sets were removed if they were located on any contig that was flagged from the alien index (AI) pipeline. This resulted in a final gene set of 68,514 genes. All intersects were performed with BEDTools.

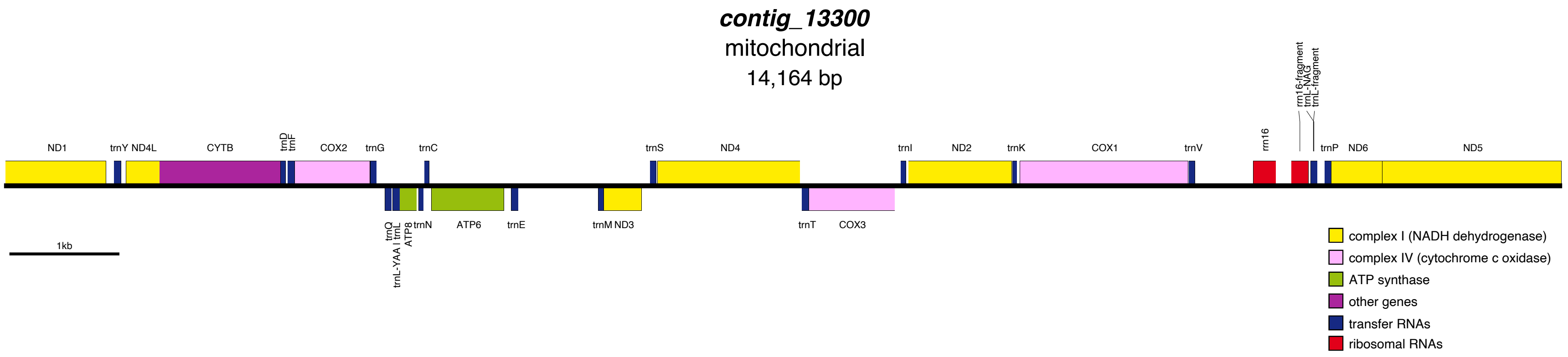

Figure S4. Physical map of mitochondrial-derived contig. Nucleotide sequences of organelle-derived contigs are available are for download from the project's FigShare data repository (see Data Availability).

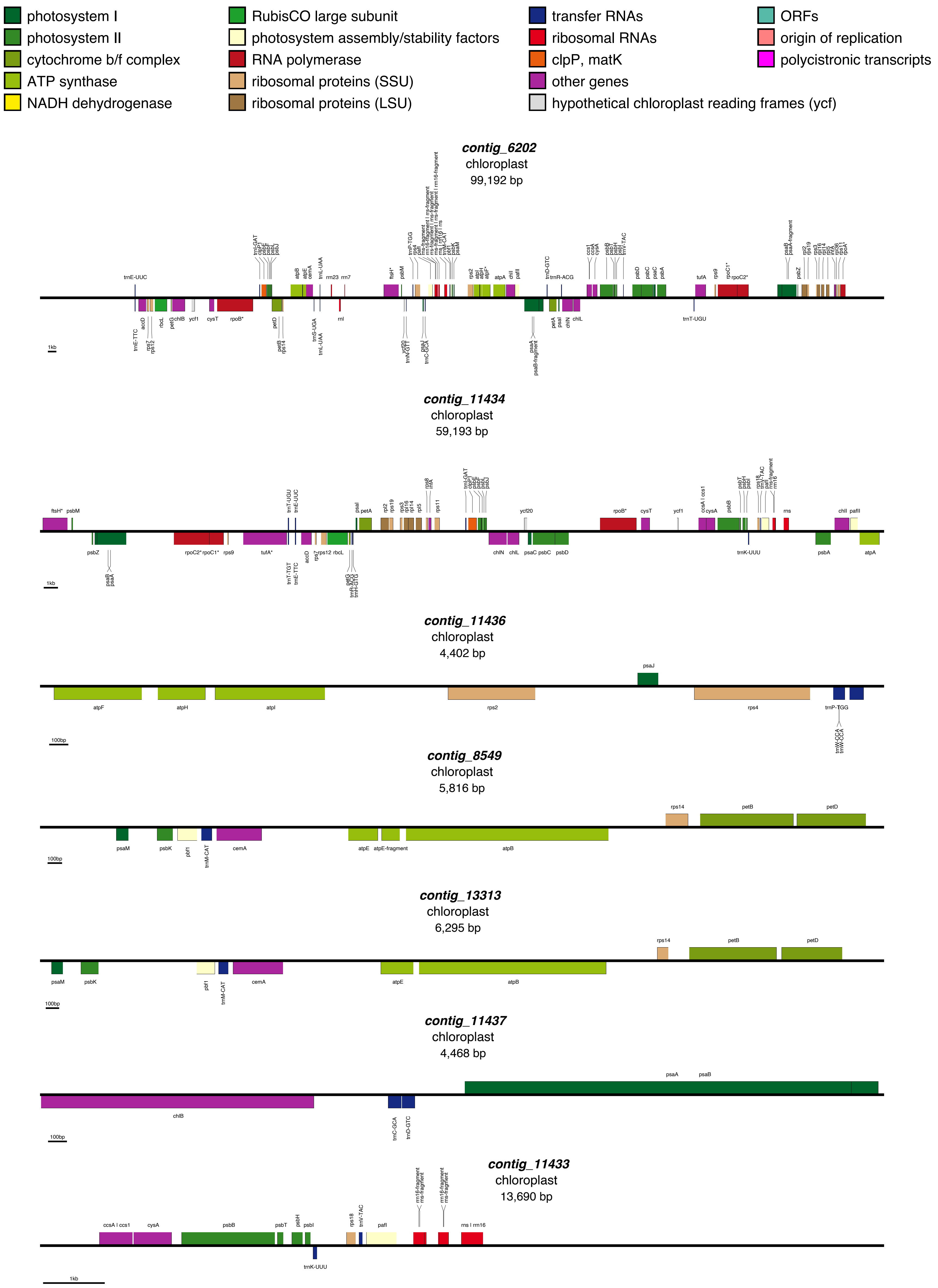

Figure S5. Physical maps of kleptoplast derived contigs. Nucleotide sequences of organelle-derived contigs are available for download from the project's FigShare data repository (see Data Availability).

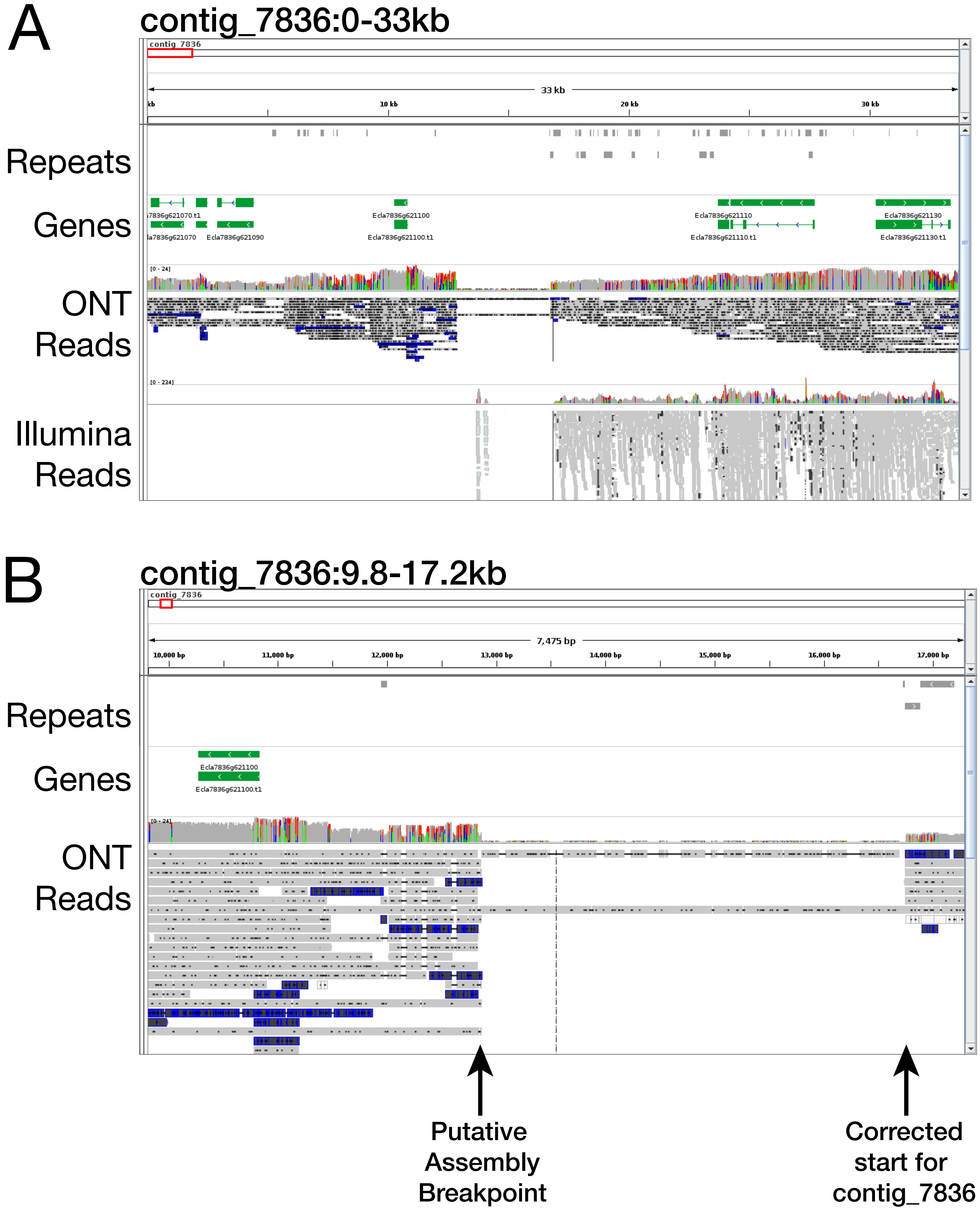

Figure S6. Read support for *PsaA/PsaB* locus on contig_7836. Illumina and ONT read alignments relative to genes and repetitive elements are visualized along the proximal end of contig_7836 in these IGV screenshots. In each figure, the read depths and alignments are provided as histograms and horizontal bars, respectively. The entire locus assembled from possible contamination is observed in (A), whereas a zoomed in view of the distal end of misassembled locus is in (B). Highlighted in (B) is the putative breakpoint of the presumed misassembly at ~12.8kb, as well as the corrected start site used for contig_7836 in the final assembly. ONT reads colored blue have non-canonical alignments which are indicative of possible indels.

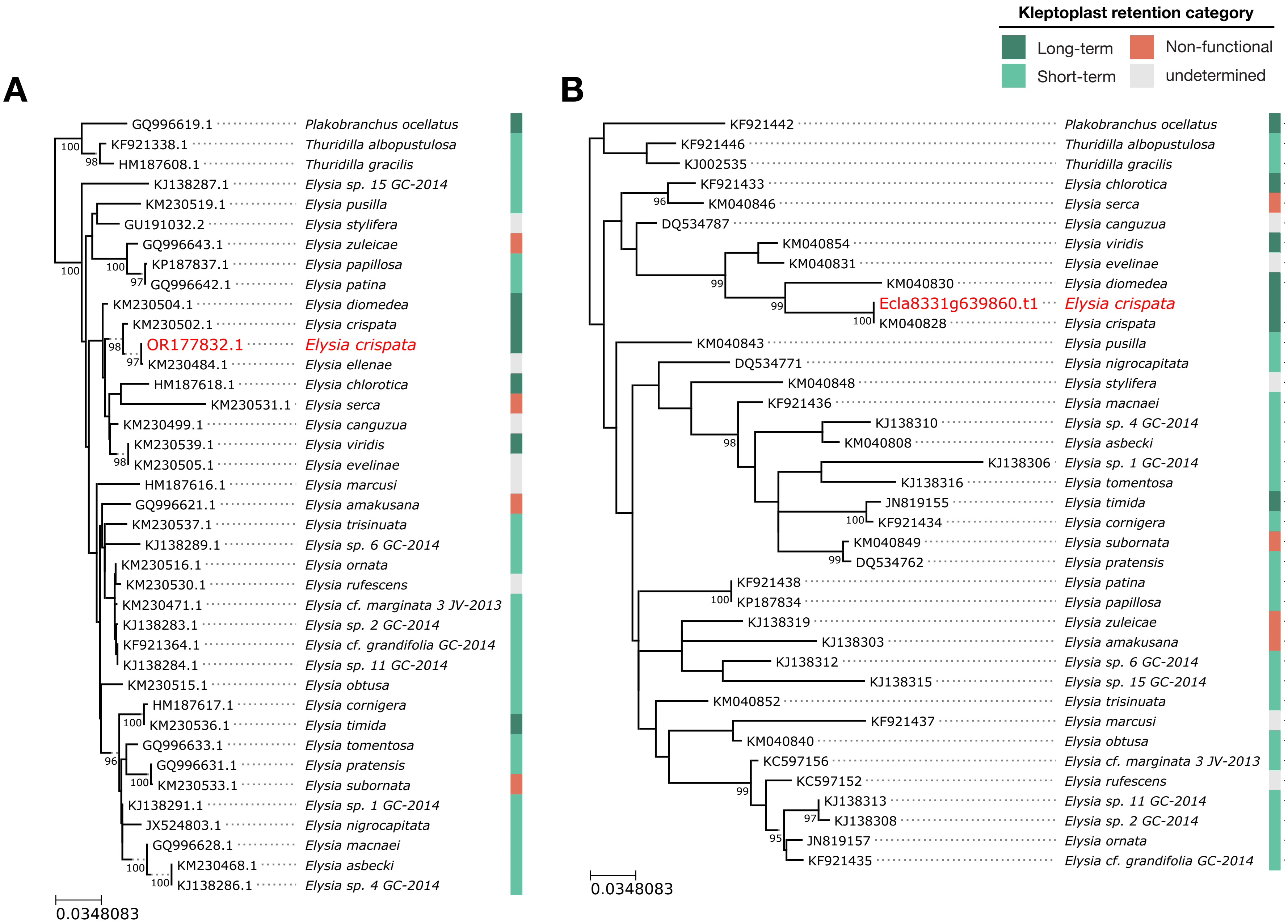

Figure S7. Phylogenetic analysis of sacoglossan sea slugs. Maximum likelihood trees of 28S (A) and H3 (B) loci showing the relationship of *E. crispata* genome sequences (red) to other kleptoplastic sea slugs. Trees were rooted on the *Thuridilla/ Plakobranchus* clade. Numbers along select branches indicate IQ-TREE ultrafast bootstrap support values (≥ 95) for the descendant nodes. The color bar indicates the kleptoplast retention state for each species: long-term retention (>20 days); short-term retention (>1 day and <20 days); see Table S12 for references.

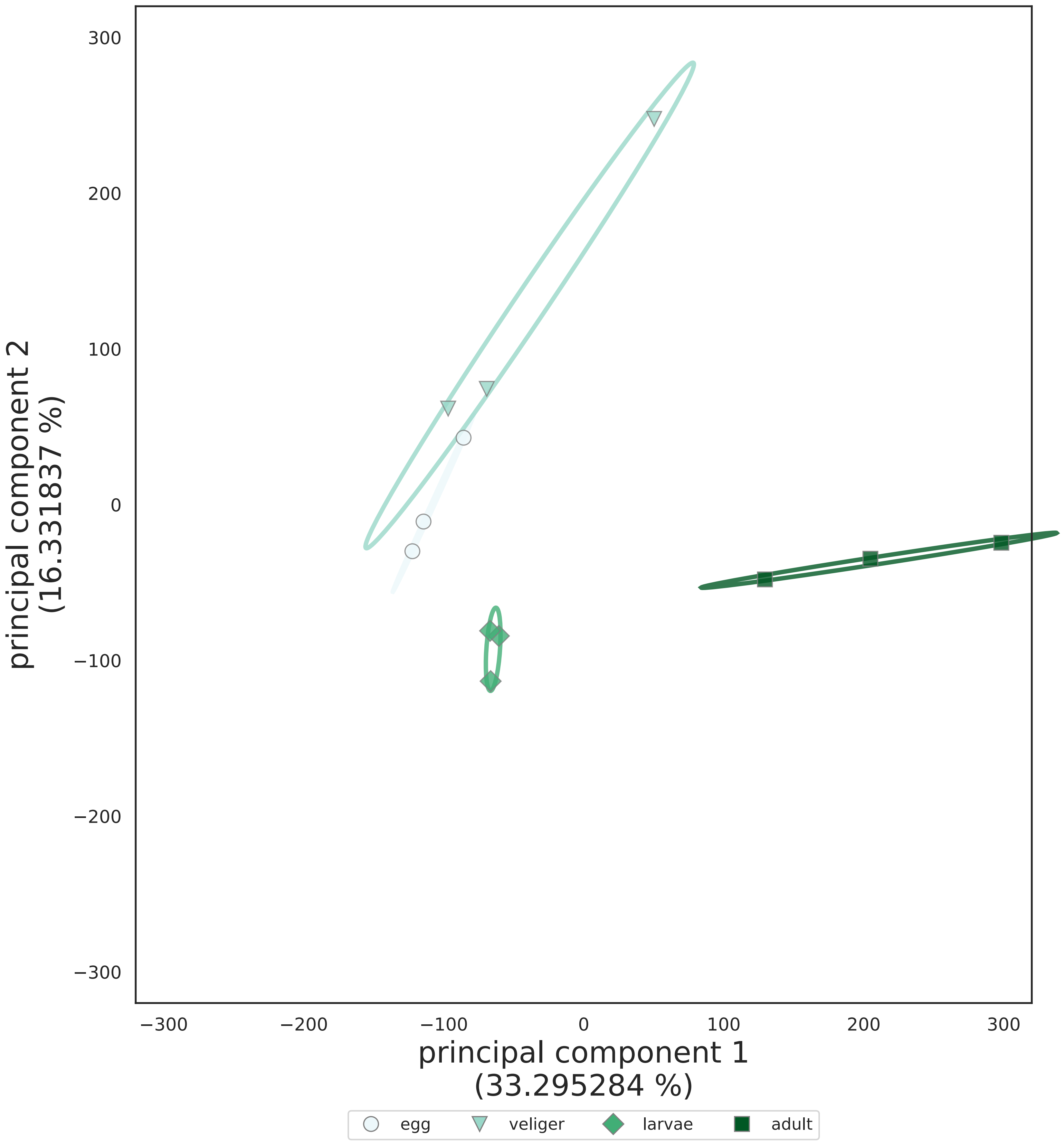

Figure S8. PCA plot depicting first two principle components of RNAseq analysis from four *E. crispata* developmental life stages.

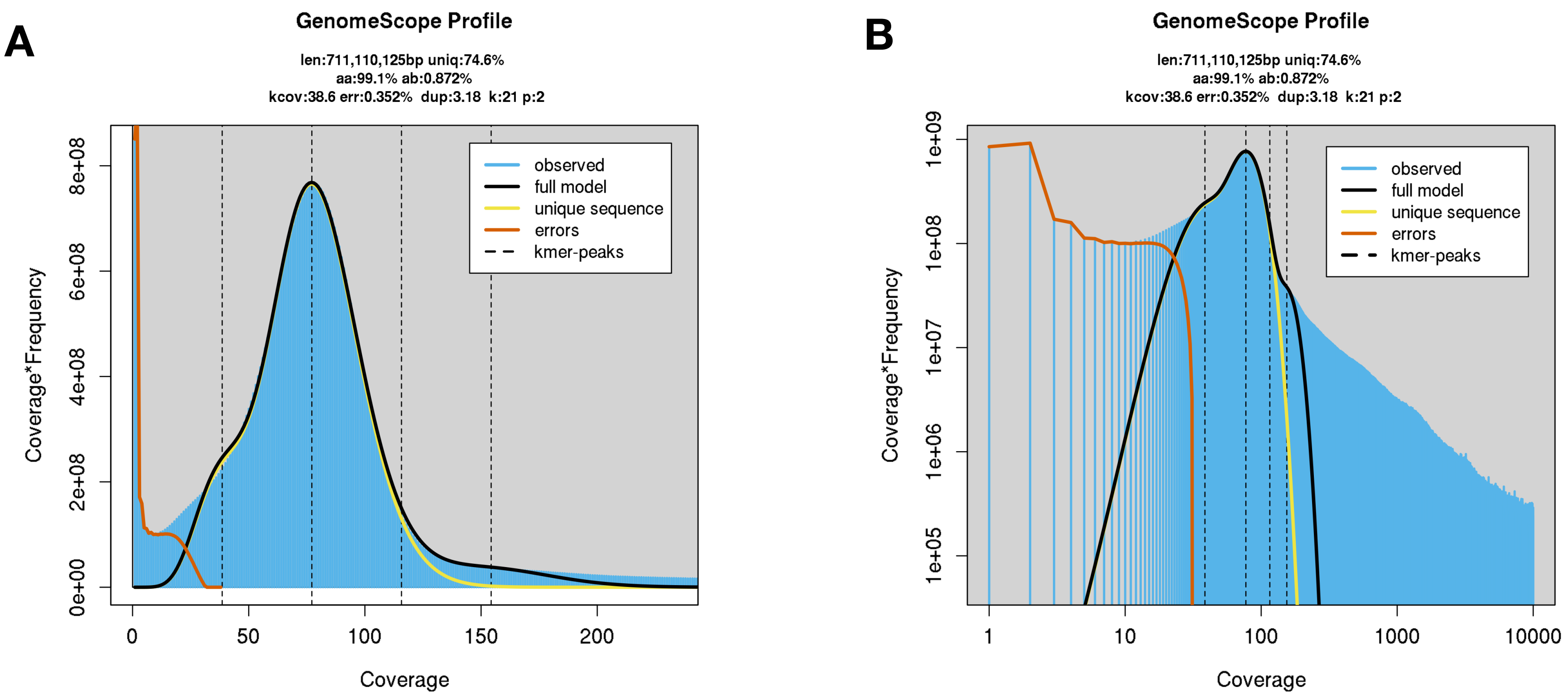

C

Property	min	max
Homozygous (aa)	99.12%	99.14%
Heterozygous (ab)	0.86%	0.88%
Genome Haploid Length	709,349,889 bp	711,110,125 bp
Genome Repeat Length	179,856,906 bp	180,303,217 bp
Genome Unique Length	529,492,983 bp	530,806,908 bp
Model Fit	77.24%	97.45%
Read Error Rate	0.35%	0.35%

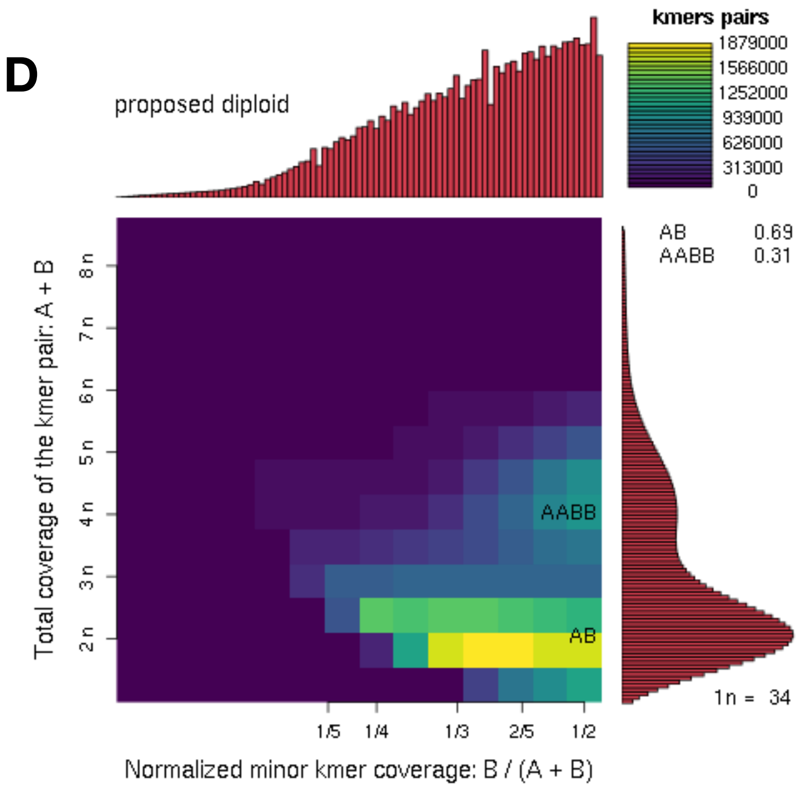

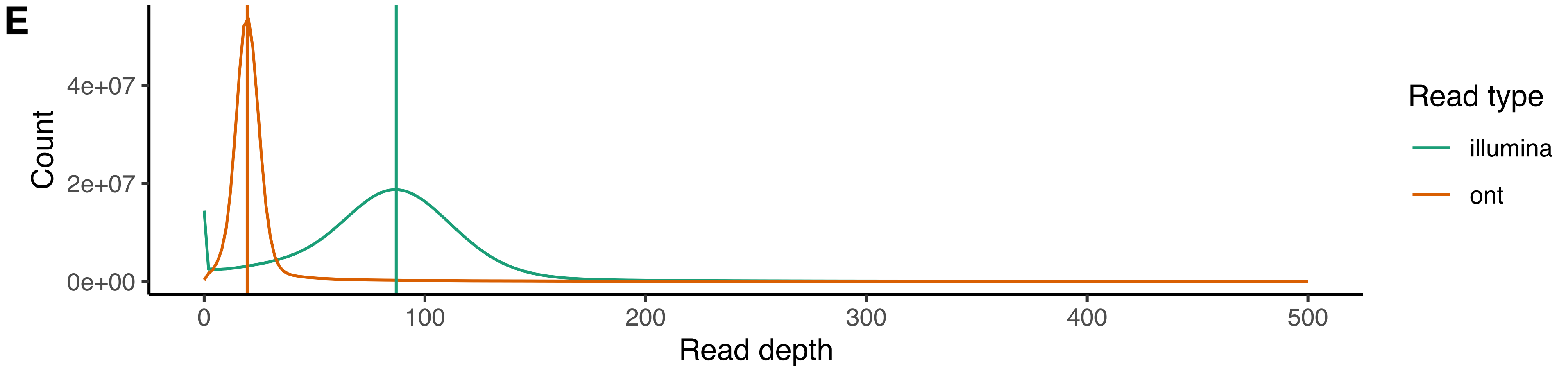

Figure S9. Summery of genome coverage statistics. GenomeScope2.0 k-mer profile plot of the *E. crispata* ECLA1 illumina gDNA reads depicted with a linear (A) and log transformed (B) x-axis. The blue bars indicate the observed kmer frequencies, and the black and yellow lines indicate expected distribution of kmers using the GenomeScope model for diploid genomes. Reads were filtered using BlobPlot prior to the GenomeScope analysis. C) GenomeScope2.0 Summary statistics. D) Smudge plot of same reads used in GenomeScope analysis indicating *E. crispata* ECLA1 is a diploid organism. E) Read-depth histograms of the *E. crispata* genome displaying coverage of ONT nanopore reads (orange) and illumina gDNA reads (green). Vertical bars indicate the maximal read depth for each distribution; 20 for ONT and 86 for Illumina.
